# Pityriasis lichenoides: assessment of 41 pediatric patients

**DOI:** 10.1016/j.jped.2024.03.011

**Published:** 2024-04-24

**Authors:** Aluhine L. Fatturi, Mariana A.P. Morgan, Jandrei R. Markus, Lucero Noguera-Morel, Vânia O. Carvalho

**Affiliations:** a Pediatric Dermatology Division, Department of Pediatrics, Federal University of Paraná, UFPR, Curitiba, Brazil; b Department of Dermatology, Hospital Infantil Universitario Niño Jesus, Madrid, Spain

**Keywords:** Child, Clinical diagnosis, Pityriasis lichenoides, Treatment

## Abstract

**Objectives:**

This study aims to evaluate the characteristics and treatment response of patients with pityriasis lichenoides seen in the last 43 years in a pediatric dermatology service.

**Methods:**

This was a retrospective, analytical, longitudinal study of patients under 15 years of age. The medical records were reviewed and data were presented as frequencies, means and variances. Student's t-test, Mann-Whitney test, Fisher's exact test, Pearson/Yates chi-square test and multivariate logistic regression model were used, with *p* < 0.05 considered.

**Results:**

41 patients were included, 32 (78.0%) with pityriasis lichenoides chronica (PLC), five (12.2%) with pityriasis lichenoides et varioliformis acuta (PLEVA) and four (9.8%) with clinical PLC without biopsy. The age range of school children and adolescents was 19 (46.3%) and 13 (31.7%) respectively and 27 (65.8%) were male. Two peaks of the highest frequency were observed between 2004 and 2006 (10 patients - 24.4%) and another between 2019 and 2021 (6 patients - 14.7%). There was remission in 71.9% (*n* = 23), with 56.6% (*n* = 17) of those who used antibiotic therapy and 80% (*n* = 4) of those who had phototherapy. The chance of remission was 13 times greater in patients with disease onset after 5 years of age.

**Conclusions:**

The clinical form most commonly found was PLC mainly in school children and adolescents. The frequency peaks coincided with infectious outbreaks. The remission rate was satisfactory with antibiotic therapy, but higher with phototherapy. Remission was greater in patients with disease onset after 5 years of age.

## Introduction

Pityriasis lichenoides (PL) is an inflammatory dermatitis that mainly affects children and young adults.^1^^,^^2^ Its prevalence, incidence, and risk factors are yet to be defined.^3^

Several hypotheses have been raised about its pathophysiology. It is considered to be a lymphoproliferative disease, possibly triggered by antigenic stimuli, mainly viruses and drugs,^4^^,^^5^ representing the benign spectrum of cutaneous lymphoproliferative disorders.^6^

Few epidemiological studies are available and the number of pediatric patients included is even smaller.^7^, ^8^, ^9^

Clinical forms include pityriasis lichenoides et varioliformis acuta (PLEVA), febrile ulceronecrotic Mucha-Habermann disease (a subtype of PLEVA associated with fever), and pityriasis lichenoides chronica (PLC). More than one type can coexist in the same patient.^2^ The acute form tends to be self-limiting and is characterized by generalized papules, which progress to necrosis, and a varioliform scar may remain.^10^ The chronic form is the most common, with periods of remission and recurrence over several months or years. It is characterized by erythematous to brownish papules and plaques, with peeling from the periphery to the center of the lesion (Mica-like scale),^1^ and evolving into hypochromic patches without scarring.^11^ The distribution of lesions is usually central, preferentially affecting the trunk and proximal extremities.^7^

The literature regarding treatment in children is scarce but oral antibiotics (such as erythromycin and tetracycline), phototherapy, topical corticosteroids, and immunosuppressants have been reported as options.^4^^,^^7^

The objectives of this study were to evaluate the clinical characteristics, time for diagnosis, triggering factors and response to treatment of patients with PL seen over the last 43 years in a tertiary service in Southern Brazil.

## Methods

### Study design and population

A retrospective cross-sectional study was conducted in the pediatric dermatology department of a university hospital in Southern Brazil from 1980 to 2023.

For data analysis, four decades were compared: 1980–1989, 1990–1999, 2000–2009 and 2010–2019, as were the four seasons.

### Variables

Medical records of patients under 15 years of age diagnosed with PL were reviewed using a protocol prepared for the study to assess sex, age at symptom onset, age at diagnosis, time to diagnosis, season of the year at symptom onset, triggering factors, duration of the disease, characteristics and distribution of lesions, associated symptoms, histopathological findings, treatment used, treatment time, and response to treatment. The photographs of the included patients were reviewed.

### Statistical analyses

The variables collected were analyzed using descriptive statistics with frequency distribution for categorical data and median and interquartile range for asymmetric continuous data.

The Pearson/Yates chi-squared test was used to compare categorical variables. The multivariate logistic regression model, considering remission for more than six months or the permanence of skin lesions as the dependent variable, was used to identify the most predictive variables and their respective odds ratio (OR).

The sample confers 95% testing power considering the type II error of 5%, type error of 5% and estimated prevalence of 3%. Statistical significance was deemed present in p-values less than 0.05. The data were analyzed with the *Statistica*® program (StatSoft Power Solutions, Inc., Palo Alto, California, USA).

### Ethics statements

The study was approved by the institution's Research Ethics Committee, under No. 69842723.1.0000.0096. All patients sign the consent form.

## Results

### Population description

Of the 43 years of the study time, the authors evaluated 41 patients with PL: 37 (90%) confirmed by biopsy, of which 32 (78.0%) had PLC and five (12.2%) PLEVA. Four (9.8%) patients without biopsy, but with a clinical presentation compatible with PLC. On average, each patient had four images of lesions, which allowed us to confirm the description in the medical records.

Table 1 presents the patients’ characteristics.

**Table 1:** Characteristics of patients, frequency of skin lesions in all patients and frequency of skin lesions according to age group.

Characteristics			n (%)/median (IQR)	
Sex				
Girls			14 (34.2%)	
Boys			27 (65.8%)	
Age group				
Infants			1 (2.4%)	
Preschool children			8 (19.5%)	
School children			19 (46.3%)	
Adolescents			13 (31.7%)	
Age at medical consultation (years old)			9 (6–12)	
Age at disease onset (years old)			8 (4–10)	
Time to diagnosis (months)			5.0 (2–120)	

Types of lesions*			n (%)	

Papule			38 (92.7%)	
Peeling			30 (73.2%)	
Hypopigmentation			30 (73.2%)	
Annular lesion			25 (61.0%)	
Erythema			22 (53.6%)	
Crust			17 (41.5%)	
Hyperpigmentation			7 (17.1%)	
Necrosis			4 (9.7%)	

Skin lesions	Preschool children (*n* = 8)	School children (*n* = 19)	Adolescents (*n* = 13)	p**

Papule	7 (87.5%)	18 (94.7%)	12 (92.3%)	0.80
Peeling	7 (87.5%)	14 (73.7%)	9 (69.2%)	0.63
Hypopigmentation	5 (62.5%)	13 (68.4%)	12 (92.3%)	0.20
Annular lesion	6 (75.0%)	12 (63.2%)	6 (46.1%)	0.39
Erythema	5 (62.5%)	11 (57.9%)	5 (38.5%)	0.45
Crust	5 (62.5%)	6 (31.6%)	5 (38.5%)	0.32
**Hyperpigmentation**	**3 (37.5%)**	**4 (21.0%)**	**0 (0.0%)**	**0.07**
Necrosis	0 (0.0%)	2 (10.5%)	1 (7.7%)	0.63

⁎ All patients had more than one type of lesion.

⁎⁎ Pearson's chi-square test.

### Prevalence over the decades and seasons

**Figure 1:**
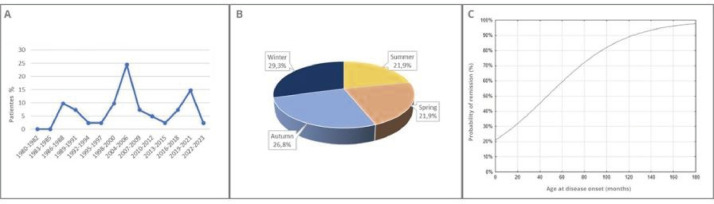
Frequency distribution of pityriasis lichenoides. (A) Distribution from 1980 to 2023. (B) Distribution according to the seasons of the year. (C) Probability of remission of pityriasis lichenoides according to the age at disease onset.

Figure 1A shows the prevalence distribution of PL over the 43-year study period, with two peaks of higher frequency, one from 2004 to 2006 (10 patients - 24.4%) and another from 2019 to 2021 (6 patients - 14.7%).

The authors found no significant difference in terms of disease onset according to the seasons of the year (*p* = 0.26; Figure 1B).

### Disease characteristics

Papules were the predominant lesion, found in 38 (92.7%) patients, followed by peeling, in 30 (73.2%) patients, hypopigmentation, also seen in 30 (73.2%) patients, and annular lesions, in 25 (61.0%) patients. A total of 22 (53.6%) and 17 (41.5%) of patients had erythema and crusts, respectively. Hyperpigmentation, necrosis, and erythematous plaques were less frequent. The types of lesions did not vary according to age group, but hyperpigmentation was more frequent in preschool children (*p* = 0.07; Table 1).

The lesions were diffuse in 19 (46.3%) cases, central in 18 (43.9%), and peripheral in four (9.7%).

In total, 18 (43.9%) patients had associated symptoms. Pruritus was the most common symptom.

Triggering factors were present in 11 (26.8%) cases, including fever (three cases), COVID-19 infection (two cases), and sun exposure, HIV, parotitis, tonsillitis, cold weather, and COVID-19 vaccination (one case each).

### Treatment features

Among the treatments used, with a median duration of 14.5 months (IQR = 5.5–48), 30 (73.2%) patients underwent antibiotic therapy, six (14.6%) corticotherapy, five (12.2%) phototherapy, and three (7.3%) topical hydration. The antibiotics administered were erythromycin in 14 (46.3%) patients and tetracycline in 11 (36.6%), and in 5 (17.1%) cases used both erythromycin and tetracycline at different times. The median number of consultations was four (IQR = 2–7), with a minimum of one and a maximum of 27.

The antibiotics used for treatment were erythromycin and tetracycline. The choice of antibiotic depended on the age of the patient (tetracycline was not used in children under eight years of age) and the availability of the medication. Of the 30 (73,1%) patients treated with antibiotic therapy, 17 (56.6%) had remission.

**Figure 2:**
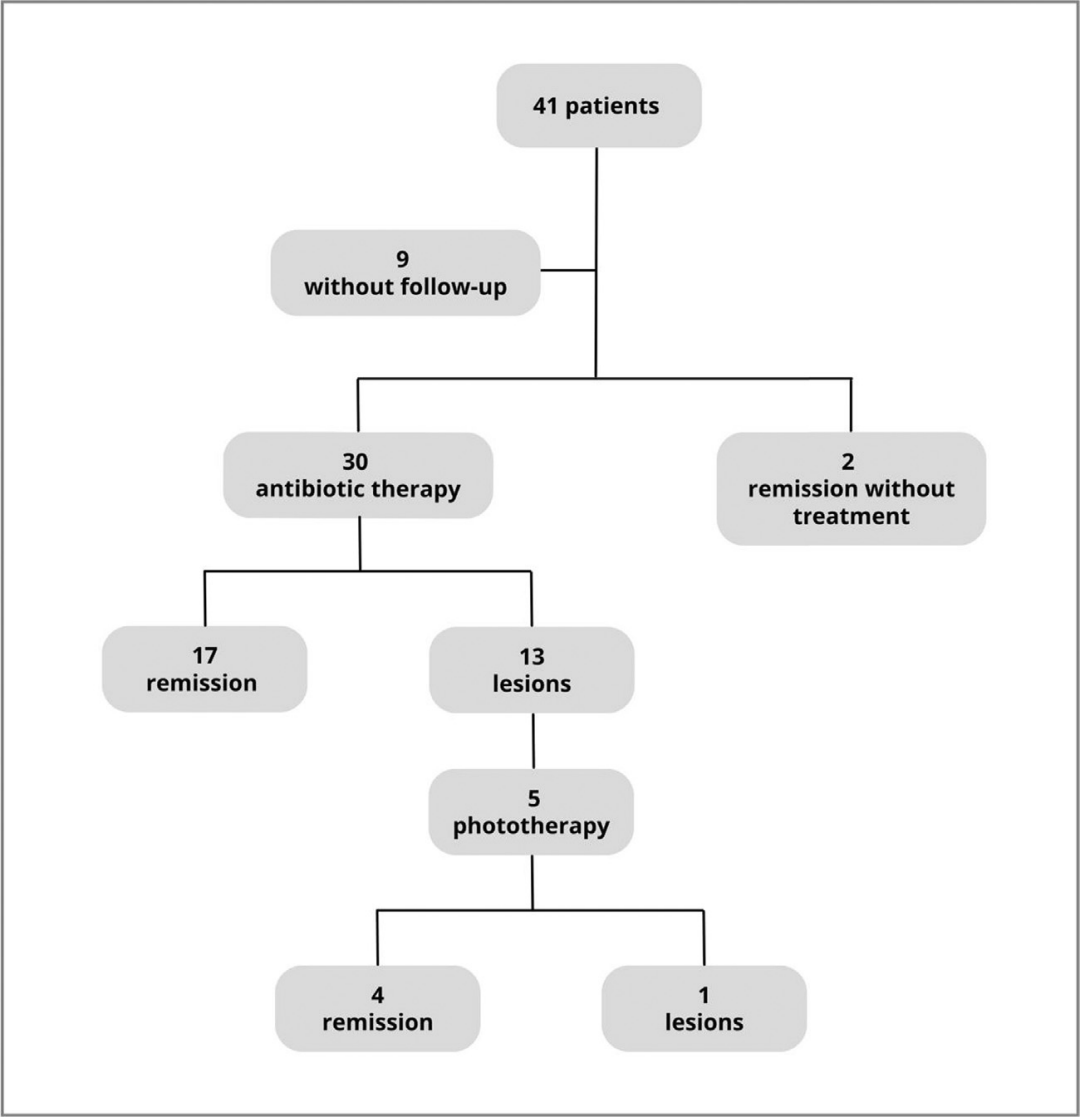
Progress of the patients evaluated according to the treatment used.

Treatment with narrowband ultraviolet B (NB-UVB) phototherapy, which was implemented at the service in 2019, was indicated according to the patient's availability to attend the sessions and in the absence of contraindications. Of the five patients treated with NB-UVB, four (80%) progressed to complete remission by the end of the study. Figure 2 shows the treatments used and the patient's progress.

### Follow-up characteristics

Of the 41 patients, nine (21,9%) did not return for follow-up. Among the remaining 32, 23 (71.9%) showed resolution of the disease and nine (28.1%) remained symptomatic at the last evaluation.

The absence of lesions for more than six months at the last evaluation was observed more frequently among school children (12 patients - 54.5%) and adolescents (9 patients - 40.9%) (*p* < 0.001).

In the multivariate logistic regression model, considering remission for more than six months or permanence of skin lesions as the dependent variable, only the time of disease onset showed a significant association, with 13 times greater chances of remission with disease onset after five years of age (*p* = 0.01) (OR = 13.33; 95% CI = 2.11–84.13) (Table 2).

**Table 2:** Strength of association between the variables studied and remission of pityriasis lichenoides.

Variables	OR*	95% CI**	p
Sex/Gender	0.76	0.04–10.07	0.76
Age group	0.80	0.08–7.16	0.84
**Age at disease onset**	**13.33**	**2.11–84.13**	**0.01**
Time to diagnosis	0.40	0.05–2.94	0.37
Season of the year	0.75	0.26–2.19	0.60
Histology	1.87	0.13–26.19	0.64
Worsening factors	2.48	0.09–62.77	0.58
Treatment	3.6	0.78–16.62	0.09
Treatment time	1.00	0.95–1.06	0.82
Location of lesions	2.52	0.43–14.6	0.30

⁎ OR, odds ratio.

⁎⁎ CI; interquartile range.

The univariate logistic regression model also showed that the later the disease onset, the higher the estimated probability of remission (Figure 1C).

## Discussion

Although some clinical findings are characteristic of PL, its diagnosis is challenging and therefore sometimes delayed. In this study, the mean time from symptom onset to diagnosis was five months, with a maximum of 10 years. A previous series from The Johns Hopkins Hospital in the United States demonstrated a mean time from symptom onset to diagnosis of one year, varying up to a maximum of three years, a mean age at onset of 8 years old as in the present study with a slight male predominance also in concordance with these findings.^7^

The most frequent clinical form of the disease was PLC (78%), corroborating Khachemoune in a review on the topic^1^ and Zang and Romaní in epidemiological studies.^7^^,^^9^ The clinical presentation of PLC includes papules, desquamation, hypopigmentation, and annular lesions,^1^^,^^2^^,^^7^ which were also the most frequent skin lesions in this study.

The lesions were mainly diffusely distributed in 19 (46.3%) patients, similar to the study by Romaní in Spain, which retrospectively evaluated 22 pediatric patients with PL and found that 64.2% had diffusely distributed lesions.^9^

Regarding associated symptoms, Ersoy-Evans, in a retrospective analysis of 124 pediatric patients, observed that 85.7% were symptomatic and 62% of the symptoms were pruritus.^10^ In this study, most patients 25 (56.1%) were asymptomatic, but when present, the most prevalent symptom was also pruritus.

Triggering factors, such as fever, tonsillitis, parotitis, COVID-19 infection, and COVID-19 vaccination, were correlated with infectious diseases. Ersoy-Evans et al. identified infectious agents preceding disease onset in 30% of cases.^8^ In the study by Durusu et al., the onset of PL in a 42-year-old patient was 10 days after COVID-19 infection.^5^ This supports the etiological hypothesis of lymphoproliferative disorders possibly triggered by antigenic stimuli, such as viruses or other infectious agents.^5^ Another important finding of this study that corroborates this hypothesis was the peak incidence from 2004 to 2006, corresponding to a peak of viral meningitis that occurred in Southern Brazil in this period,^12^ and from 2019 to 2021, coinciding with the COVID-19 pandemic and its period of greatest contagion.^13^

Both Ersoy-Evans and Zang observed a seasonal characteristic of PL, with worsening in fall and winter. Although this study found a slightly higher prevalence in fall (11 patients - 26.8%) and winter (12 patients - 29.3%), this difference was not statistically significant and thus the authors could not affirm the existence of a seasonality for the disease.

The remission rate in this study was 71.9% (*n* = 23). When considering each treatment in isolation, 56.6% (*n* = 17) of patients treated with antibiotic therapy and 80% (*n* = 4) of patients treated with NB-UVB phototherapy had remission. These data are similar to the findings of Jung et al. in a systematic review of the treatment of PL with 27 articles, in which remission with antibiotic therapy ranged from 53 to 66% and with NB-UVB phototherapy was 75%.

Remission was greater the later the disease onset, up to 13 times greater when PL started after five years of age. Bellinato showed this in a systematic review of PL treatments, including 37 articles, in which pediatric patients had worse response rates to the proposed treatments compared with adults.^4^

One of the main limitations of this study is its retrospective design and the lack of follow-up for nine of the 41 patients, besides the 4 patients without skin biopsy to confirm the diagnosis. Longer follow-up of patients treated with phototherapy is also necessary in order to assess whether the rate of remission is maintained.

In conclusion, PL is a rare disease, which makes its diagnosis challenging and late. Infectious agents should be considered important triggers of the disease. Remission was greater in patients with disease onset after five years of age and, although the response to antibiotic therapy was satisfactory, the response to phototherapy was superior.

## Funding sources

No funding was received to assist in the preparation of this manuscript.

## Conflicts of interest

Aluhine Lopes Fatturi, Mariana Aparecida Pasa Morgan, Lucero Noguera Morel and Jandrei Rogerio Markus has nothing to declare. Vânia Oliveira de Carvalho has been an investigator of Sanofi Genzyme and speaker for Expanscience, Johnson & Johnson Consumer Health, Galderma, Mantecorp, Megalabs and Royal Canin.
